# N-Acetylcysteine Compared to Metformin, Improves The Expression Profile of Growth Differentiation Factor-9 and Receptor Tyrosine Kinase c-Kit in The Oocytes of Patients with Polycystic Ovarian Syndrome

**DOI:** 10.22074/ijfs.2018.5142

**Published:** 2017-10-14

**Authors:** Ebrahim Cheraghi, Malek Soleimani Mehranjani, Seyed Mohammad Ali Shariatzadeh, Mohammad Hossein Nasr Esfahani, Behrang Alani

**Affiliations:** 1 Department of Biology, Faculty of Sciences, University of Qom, Qom, Iran; 2 Department of Biology, Faculty of Science, Arak University, Arak, Iran; 3 Department of Reproductive Biotechnology, Reproductive Biomedicine Research Center, Royan Institute, ACECR, Isfahan, Iran; 4 Department of Applied Cell Science, Faculty of Medicine, Kashan University of Medical Sciences, Kashan, Iran

**Keywords:** Gene Expression, Metformin, N-acetylcysteine, Oocyte, Polycystic Ovarian Syndrome

## Abstract

**Background:**

Paracrine disruption of growth factors in women with polycystic ovarian syndrome (PCOS) results in production of low quality oocyte, especially following ovulation induction. The aim of this study was to investigate the effects of metformin (MET), N-acetylcysteine (NAC) and their combination on the hormonal levels and expres- sion profile of GDF-9, BMP-15 and c-kit, as hallmarks of oocyte quality, in PCOS patients.

**Materials and Methods:**

This prospective randomized, double-blind, placebo controlled trial aims to study the effects of MET, NAC and their combination (MET+NAC) on expression of GDF-9, BMP-15 and c-kit mRNA in oocytes [10 at the germinal vesicle (GV) stage, 10 at the MI stage, and 10 at the MII stage from per group] derived following ovulation induction in PCOS. Treatment was carried out for six weeks, starting on the third day of previous cycle until oocyte aspiration. The expression of GDF9, BMP15 and c-kit were determined by quantitative real time polymerase chain reaction (RT-qPCR) and western blot analysis. Data were analyzed with one-way ANOVA.

**Results:**

The follicular fluid (FF) level of c-kit protein significantly decreased in the NAC group compared to the other groups. Significant correlations were observed between the FF soluble c-kit protein with FF volume, androstenedione and estradiol. The GDF-9 expression in unfertilized mature oocytes were significantly higher in the NAC group com- pared to the other groups (P<0.001). Similar difference was not observed between the MET, NAC+MET and control groups. The c-kit expression in unfertilized mature oocytes were significantly lower in the NAC group compared to the other groups (P<0.001). Similar difference was not observed between the MET, NAC+MET and control groups (Registration number: IRCT201204159476N1).

**Conclusion:**

We concluded that NAC can improve the quality of oocytes in PCOS.

## Introduction

Anovulation associated with polycystic ovary syndrome (PCOS), as a common metabolic disorder, is the major cause of female infertility (1,2). The principal feature of PCOS is the large number of follicles arresting at early growth stage. The cytoplasmic and nuclear maturity of oocytes is reduced following ovarian stimulation and may account for embryo quality in these couples (3). Exclusive oocyte secreted factors (OSFs), such as growth differentiation factor-9 (GDF-9) and bone morphogenetic factor-15 (BMP-15), belonging to for oocyte competence (3,5). The receptor tyrosine kinase c-kit is another OSFs which plays important role in oogenesis and folliculogenesis (6). Recent evidence suggest possible involvement of c-kit and its receptor, kit ligand (KL) in PCOS pathology (7). Indeed, it has been shown that the aberrant or low expression of these exclusive oocyte secreting factors (BMP-15 and GDF-9), lead to over expression of c-kit and its receptor (8-10). Therefore, drugs which can modulate the regulation of these intra-ovarian factors may play a role in the clinical management of PCOS. 

To improve the quality of oocyte, various protocol tion induction along with insulin-sensitizing drugs in PCOS patients. But, the risks of poor response, ovarian hyperstimulation, production of low quality oocytes, reduced fertilization rates and poor embryo quality remains among concern to be dealt within PCOS women undergoing *in vitro* fertilization (IVF) or intracytoplasmic sperm injection (ICSI) (11). Metformin, an insulin-lowering agent, has been extensively used for treatment of anovulation and infertility in PCOS patients. ed (12). In this regard, background studies indicate that MET does not improve the overall outcomes of assisted reproductive procedure in term of the aforementioned parameters (13,14). 

On the contrary, administration of N-acetylcysteine (NAC) has been shown to improve not only the number and also the quality of oocytes in these patients. This phenomenon has been mainly related to the strong antioxidant effect of NAC, which has been shown to reduce follicle atresia and improve the quality of oocyte (15). *In vitro*, NAC plays a key role in cell survival through the production of trophic factor and follicular preservation (16,17). In line with these reports, Sacinositol and NAC improve ovarian function of PCOS patients. Therefore, considering the fact that oocyte secretory factors are hallmarks of oocyte quality, this study aims to evaluate the effects of NAC, MET and their co-administration on the expression of GDF-9, BMP-15 and c-kit in PCOS individuals undergoing ovarian stimulation in ICSI cycle. 

## Materials and Methods

Antibodies directed against c-kit and β-actin was obtained from Cell Signaling Technology (Beverly, MA, USA). BMP-15 antibody was obtained from Abcam Technology (Cambridge, MA, USA) and GDF-9 antibody from Santa Cruz Biotechnology (CA, USA). Other reagents used in this study were obtained from Sigma-Aldrich (St. Louis, MO, USA). Cell culture media and sera were obtained from Gibco BRL (Carlsbad, CA, USA).

### Study design

This study was performed in continuation of our prospective randomized, double-blind, placebo controlled trial, in the IVF Unit of Infertility Research Center of the Academic Center for Education, Culture and Research (ACECR), Qom/Iran. 80 infertile PCOS women at the age of 25-35 years, in the interval between July 2012 and February 2013, who planned to undergo ICSI were included in this study (19). Individuals were diagnosed as PCOS according to the Rotterdam consensus workshop (20). Based on this consensus, each individual needed to have two out of three criteria: i. Biochemical or clinical hyperandrogenism, ii. Chronic oligo or anovulation and iii. Polycystic ovaries at ultrasound examination. Ethical consideration and further information on this clinical trial are provided in previous studies (19). This study was approved by the Ethics Committee (EC/91/1041) of Royan Institute, Tehran, Iran. The patients provided an informed consent and committed to avoid any changes in their normal physical activity, diet or starting a new medical regimen throughout the study.

### Treatment design and ovulation induction

The female partner of ICSI candidates were examined and randomly divided into 4 groups (n=20): i. Placebo (PLA) receiving oral rehydration solution (ORS, Poursina, Iran), ii. MET receiving MET (Glucophage, Merck, West Drayton, UK, 500 mg), iii. NAC receiving NAC (Holzkrichen, Germany, batch no. 6N5483, 600 mg) and iv. MET+NAC group receiving the combination of MET and NAC at the aforementioned doses. Treatment was carried out three times daily for a period of six weeks. The dose and duration of NAC treatment was chosen according to recent studies (21-23).

Gonadotropin-releasing hormone (GnRH) agonist protocol (18) was used to induce ovulation. The female partner of ICSI candidates randomized to four groups received PLA, MET, NAC or MET+NAC from the third day of last menstrual period (LMP) of previous cycle until the day of oocyte aspiration. Oral contraceptive pills (OCPs) were also included in the regimen for 21 days starting simultaneously with placebo, MET, NAC or MET+NAC on the day 3 of menstrual cycle prior to the treatment cycle. For ovarian down-regulation, daily injections of Bucerelin acetate (1 mg, Suprefact, Aventis, Germany) were administered from the day 19 of the preceding, menstrual cycle until day 2 of the next cycle. On the second day on the next cycle if the endometrial thickness was less than 4 mm, the dose of Burcerelin acetate was reduced to 0.5 mg.

**Fig.1:**
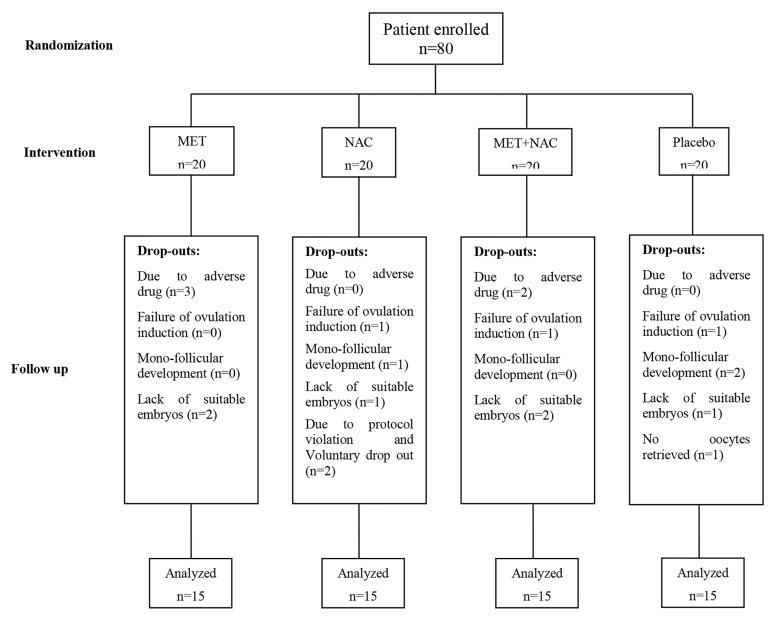
Flowchart of participants in this study. MET; Metformin, NAC; N-acetylcysteine, and PLA; Placebo.

Ovulation induction was induced from the day two of the cycle with average daily injections of 2 ampoules of recombinant follicle stimulating hormone (rFSH, Gonal-f, Merck Serono S.A., Geneva, Switzerland). Vaginal ultrasound (Honda Electronics HS 4000-Japan) was also used to monitor the cycles. 10,000 IU human chorionic gonadotropin (hCG, Pregnyl, Organon, Netherlands) was administered to induce ovulation. 36 hours after the administration of hCG, when at least three follicles had reached the diameters of 16-18 mm, transvaginal oocyte aspiration was performed under ultrasound guidance and general anesthesia. This protocol of induction ovulation was used for all the individuals in the 4 group. During the treatment the participants were asked to report any probable side effects such as abdominal discomfort, diarrhea and nausea. Due to these side effects, 20 couples (5 per group) were excluded from the study (Fig .1).

### Preparation of oocytes, follicular fluid and blood samples

Based on our pervious study, oocytes and follicular fluid (FF) from multiple follicles, from each subject were pooled as explained (18). Following oocyte retrieval, their cumulus cells were removed by exposure to 20 IU/ml hyaluronidase (ART-4007A, SAGE BioPharma, USA) in HEPES-based medium for 30 seconds followed by mechanical pipetting in HEPES-buffered HTF containing 5 mg/ml human serum albumin (ART- 3001, SAGE BioPharma, USA).

The nuclear status of each oocyte was determined under the stereo microscope (Olympus Co., Japan) and classified into three categories: i. Unfertilized mature oocyte [metaphase II (MII)] following ICSI, ii. Germinal vesicle (GV) stage, iii. Without first polar body called metaphase I (MI). For gene expression analysis, in each experimental group, 10 GV, 10 MI, and 10 MII oocytes were separately pooled and washed in phosphate- buffered saline (PBS) and transferred into RNasefree microcentrifuge tubes. 50 μl of RNAlater, RNA Stabilization Reagent (Qiagen, USA) were added to each tube and all samples were stored in a -80°C freezer until analysis. Only MII oocytes were used for ICSI.

The FF, from the first aspirated with no visible blood contamination was collect and immediately centrifuged at 3000 rpm for 10 minutes, and the supernatants were stored at -70°C for further analysis. Fasting blood sample were also collected from each participant once prior to the start of treatment (day 2 of pervious cycle) and once on the day of ovum pick up of ICSI cycle. The samples were immediately centrifuged for 10 minutes at 3000 rpm (Hettich, EBA20, UK) and the resulting serum were stored at -70°C for evaluation.

The levels of luteinizing hormone (LH, mIU/ml), FSH (mIU/ml), total testosterone (TT, ng/ml), Progesterone (ng/ml), estradiol (E2, pg/ml) and androstenedione (ng/ ml) in the FF and serum were measured in all samples using the ELISA enzyme immunoassay (Demeditec Diagnostics GmbH, Germany) according to the manufacturer’s protocol. The FF soluble protein level of c-Kit (pg/ml) was measured with the ELISA Kit (Abnova Corporation, Taiwan) by sandwich enzyme immunoassay technique, according to the manufacturer’s protocol.

### Gene expression analysis

Total RNA from the oocytes of each group were isolated using the EZ-10 total RNA mini-prep Kit (Bio Basic Inc., Canada), according to the manufacturer's protocol. All samples were stored at -80ºC till analysis. Complementary DNA (cDNA) was synthesized, using random hexamers [using the RevertAid First Strand cDNA synthesis Kit (Thermo scientific, USA)]. To determine the relative expression of target genes, quantitative real time polymerase chain reaction (RT-qPCR) was carried out using SYBR-Green/ ROX qPCR master mix assay (Thermo scientific, USA) by gene-specific primers (Table 1). Relative gene expression was calculated as the abundance ratio of each target gene relative to *β-actin*. The ABI step one plus (ABI, USA) instrument was used for real time PCR experiments and the ΔΔCt method for data calculation.

**Table 1:** Identity and sequence details of polymerase chain reaction (PCR) primers used to analyses mRNA expression in oocytes


Sequence primer (5´-3´)	Gene

F: CCAATAGAAGTCACCTCR: GCGATCCAGGTTAAATAGCA	GDF-9
F: CAGTCCTCTATTGCCCTTCTR: AATGGTGCGGTTCTCTCTA	BMP-15
F: ACGAATGAGAATAAGCAGAATGAAR: GAGAGGACAGCGGACCAG	c-Kit
F: GGACTTCGAGCAAGAGATGGR: AGCACTGTGTTGGCGTACAG	β-actin


Total proteins from each pool of oocytes were extracted using RIPA lysis and extraction buffer Kit (Cat No: 89900, Thermo Scientific, USA), according to the manufacturer’s instruction. Concentration of proteins was determined according to Bradford’s method using bovine serum albumin (BSA) as a reference standard (Bradford, 1976). Total proteins were electrophoresed in 12.5% sodium dodecyl sulfate polyacrylamide gel electrophoresis (SDS-PAGE) gel, transferred to polyvinylidene fluoride membranes, and probed with specific antibodies. Membranes were developed using enhanced chemiluminescence reagents (Amersham Bioscience, USA) and the intensity of immunoreactive polypeptides was analyzed subsequent to visualization of the bands developed on a photographic film. Protein bands on photographic film were quantified by densitometry scanning after background subtraction. Integrated densities of bands were measured by Image J software.

### Statistics

The normality of continuous variables was confirmed using the Kolmogrov-Smirnov test and data were reported as means ± SEM. Data analysis were performed using one-way ANOVA and Tukey’s test for post-hoc. Means were considered significantly different at P<0.05. Pearson’s correlation test defined the relation between variables. All data were analyzed with the statistical software SPSS (version16.0 for windows, Chicago, IL, USA).

## Results

Patients characteristics including age, body mass index (BMI), the level of LH, FSH, E2 and TT were not significantly different among the groups PCOS prior to treatment.

### Follicular fluid analysis

**Fig.2:**
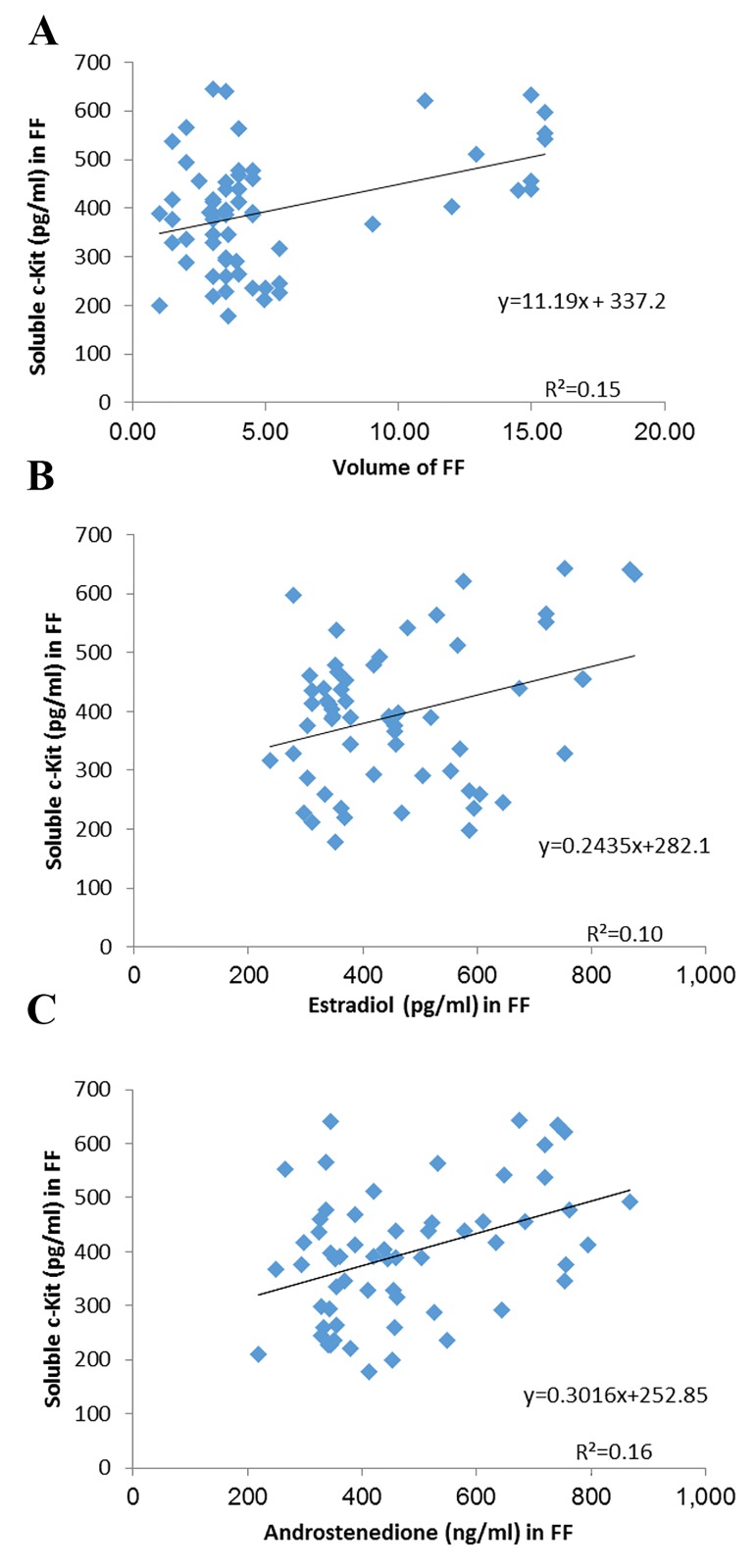
Correlation between parameters of follicular fluid (FF) in all the population. A. Soluble c-Kit with volume of FF, B. Estradiol (E2), and C. Androstenedione. R_2_; Determination of coefficient.

FF volume and FF level of androstenedione, E2 and progesterone were similar in all groups (P>0.05, Table 2), but the level of soluble c-kit protein in the FF significantly decreased in the NAC group compared to other groups (P<0.01). Our results also showed a significant correlation between the soluble c-kit protein in the FF of all the population with the FF volume (r=0.508, P=0.02), androstenedione (r=0.682, P=0.01), and E2 (r=0.638, P=0.01) (Fig .2).

### Evaluation of oocyte and embryo quality

**Fig.3:**
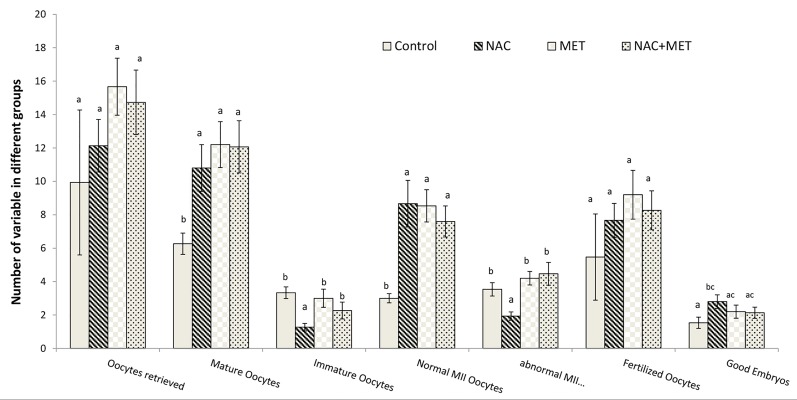
Distribution of oocytes retrieved, quality of oocytes and embryos in polycystic ovary syndrome patients undergoing treatment of N-acetylcysteine (NAC), metformin (MET), NAC+MET and Placebo (control). Data are the mean ± SEM. Statistical analyses were performed by ANOVA followed by Tukey’s test for multiple comparisons. Means without a common letter are significantly different (P<0.05).

The number of immature oocytes (MI+GV) and abnormal mature oocytes significantly decreased in the NAC group (P<0.01) compared to the other groups. Similar reduction was also observed in MET and MET+NAC groups but the reduction was not significant compared to the placebo group (P>0.05). The fertilization rate of metaphase II oocytes were similar in all groups (P>0.05). The number of good embryo (grade I) on day 3 showed a significant increase in the NAC group (P<0.02) compared to placebo group. This improvement was also observed in the MET and MET+NAC groups when compared to the placebo (P>0.05), but remained insignificant (Fig .3). The percentage of top grad embryos was not different between the three NAC with MET and MET+NAC groups.

**Table 2:** Comparison of the biochemical parameters of follicular fluid in PCOS patients


PlA	NAC+MET	MET	NAC	Parameter

Follicular fluid Volume (ml)	5.4 ± 1.13^a^	5.7 ± 1.1^a^	4.96 ± 1.1^a^	4.9 ± 1.1^a^
Estradiol (pg/ml)	466.6 ± 34.8^a^	496.6 ± 44.8^a^	470.2 ± 48.4^a^	436 ± 40.16^a^
Progesterone (ng/ml)	3983.7 ± 353.9^a^	3916.9 ± 359.5^a^	3501.2 ± 326.9^a^	3255.3 ± 414.8^a^
Androstenedione (ng/ml)	426.2 ± 30.3^a^	435.2 ± 46.7^a^	487.6 ± 42.9^a^	548.3 ± 42.36^a^
Soluble c-Kit (pg/ml)	317.8 ± 27.5^b^	380.8 ± 30.3^a^	429.8 ± 28.7^a^	455.2 ± 28.75^a^


Data are shown as mean ± SEM. Analysis was performed by ANOVA and Tukey’s test for multiple comparisons. Means without a common letter are significantly different (P<0.05). PCOS; Polycystic ovarian syndrome, MET; metformin, and NAC; N-acetylcysteine, and PLA; Placebo.

### Expression profile of GDF-9, BMP-15 and c-Kit in oocytes

**Fig.4:**
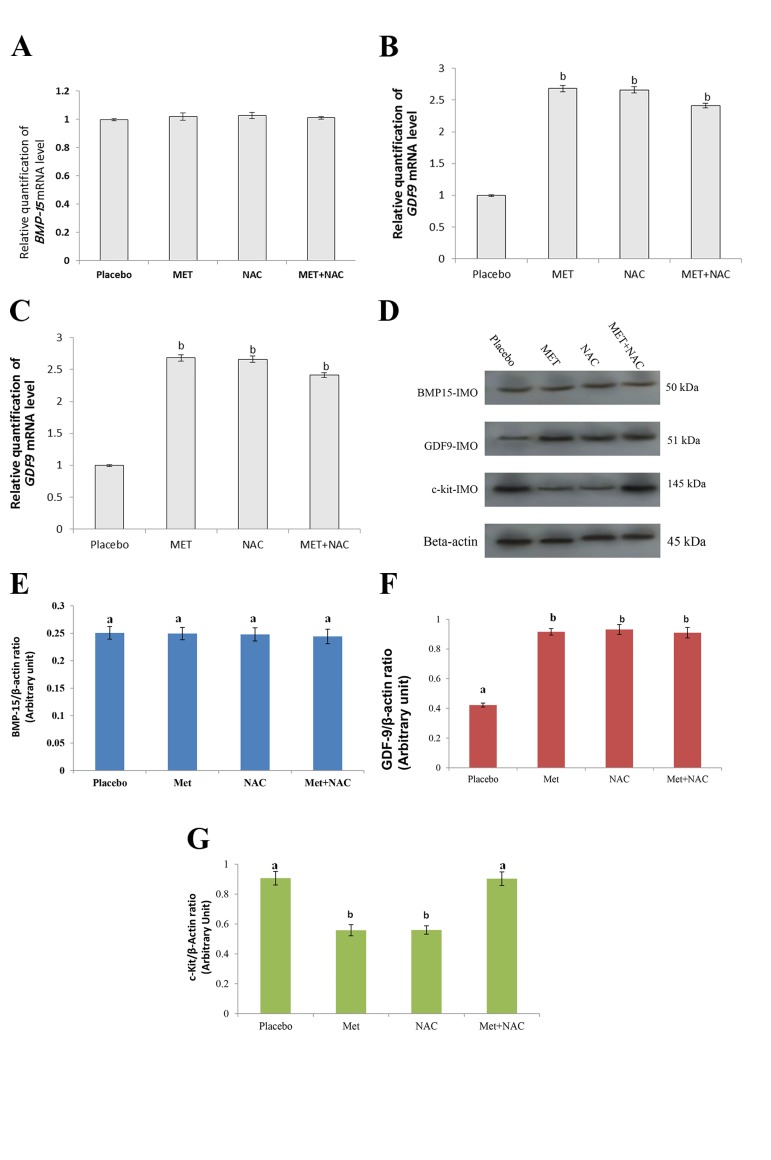
Effects of NAC and MET on *BMP-15, GDF-9* and *c-kit* mRNA and protein expression in immature oocytes (IMO, GV oocytes) of PCOS patients. Results of reverse transcriptase real-time polymerase chain reaction (PCR) for mRNAs of A. *BMP-15*, B. *GDF-9*, C. *c-kit*, in GV oocytes, D. Immunoblots of BMP-15, GDF-9 and c-kit from oocyte cell lysates. Densities of E. BMP-15, F. GDF-9 and G. c-kit protein bands in the experimental groups are shown. Means without a common letter are significantly different (P<0.05). NAC; N-acetylcysteine, MET; Metformin, GV; Germinal vesicle, and PCOS; Polycystic ovarian syndrome.

**Fig.5:**
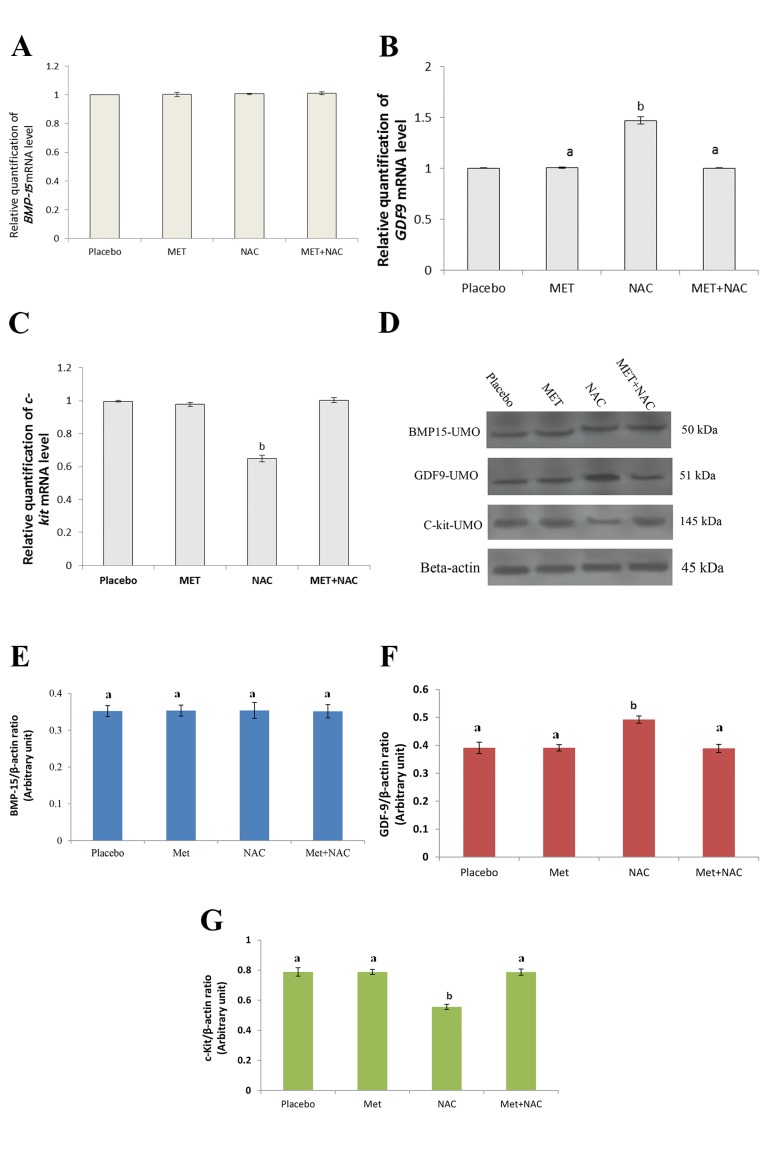
Effects of NAC and MET on *BMP-15, GDF-9* and *c-kit* mRNA and protein expression in unfertilized mature oocytes (UMO, MII oocytes) of PCOS patients. Results of reverse transcriptase real-time polymerase chain reaction (PCR) for mRNAs of A. *BMP-15*, B. *GDF-9*, C. *c-kit* in MII oocytes, D. Immunoblots of BMP-15, GDF-9 and c-kit from oocyte cell lysates. Densities of E. BMP-15, F. GDF-9, and G. c-kit protein bands in the experimental groups are shown. Means without a common letter are significantly different (P<0.05). NAC; N-acetylcysteine, MET; Metformin, and PCOS; Polycystic ovarian syndrome.

The level of BMP-15 protein in the mature unfertilized oocytes and GV oocytes did not differ significantly among the groups (P>0.05, Figes.4, 5A, D, E). The expression level of GDF-9 in the GV oocytes significantly increased in all groups compared to the placebo (P<0.001) (Fig.4B, D, F), while for unfertilized mature oocytes, GDF-9 mRNA and protein levels only significantly increased in the NAC group (P<0.001, Fig.5B, D, F). The expression of c-kit in the GV oocytes significantly decreased in the NAC and MET groups compared to the placebo group (P<0.001), but not in NAC+MET group (Fig.4C, D, G). The c-kit expression in the unfertilized mature oocytes significantly decreased in the NAC group compared to the MET and other treatment groups (P<0.001), but no significant difference was found in the MET and NAC+MET groups when compared to the placebo group (P>0.05, Fig.5C, D, G). It is important to note that the results for MI oocytes were similar to GV oocyte; therefore, in this article only the results of GV were presented. This observation is in accordance with pervious literature (24).

## Discussion

A typical characteristic of PCOS patient commonly observed during induction stimulation for ART cycles is increased number of low quality oocytes which is mainly related to state of endocrine disorder in these individuals (25). Considering the indispensable role of OSFs in oocyte development and maturation, many researches have shown impaired expression of OSFs particularly GDF-9, BMP- 15 and c-kit, may account for low quality oocyte in PCOS undergoing ovarian stimulation (26). This may explain, at least a part of the folliculogenesis disorders found in these patients (27-29). Background literature in this filed is very discrepant. Some authors have reported reduce expression of GDF-9 with no significant alteration in the expression of BMP-15 (8), while others have shown no alteration in expression of these two factors both at RNA and protein level (9) in oocyte of PCOS individuals. The exact reason of such discrepancy is not well understood.

In continue to our previous study, we demonstrated that unlike MTE and NAC+MET groups, the administration of NAC compared to placebo group, improves the maturation and quality of oocytes and also embryo development in PCOS patients undergoing ICSI (18). Therefore, in this we aimed to evaluate whether NAC could alter BMP-15, GDF-9 and c-kit levels, as the main OSFs in the oocytes of PCOS patients in comparison to MET and MET+NAC.

Compelling evidence suggest that GDF-9 and BMP-15 members of the TGFβ superfamily are exclusively expressed in the oocyte and their expression increases as follicle development progresses (30). During postnatal ovarian development, c-kit mRNA and protein are localized in the oocytes (6), and in this regard Brankin et al. (31) has shown a relation between KL/c-kit interaction with antrum formation, steroidogenesis and oocyte quality. Furthermore, genetic and descriptive studies have implicated the involvement of c-kit receptor and its ligand, KL, in oocyte growth (32).

Low GDF-9 levels is associated with abnormally increased KL level in PCOS, which could lead to abnormal ovarian features such as enlarged oocytes and increased follicle numbers (7, 32). In PCOS patients, the GDF-9 mRNA level within the oocytes is lower than in oocyte derived from normal individuals (8), and it is believed that there is a negative association between GDF-9 expression and KL/c-kit expression. Tuck (7) believes that excess androgens may act to further reduce the inhibitory effect of GDF-9, thus resulting in an abnormal increase in the KL/c-kit protein level in PCOS individual. Considering the inverse relationship between c-kit and GDF-9 in PCOS (7, 8, 32), therefore, improving the expression of GDF-9 is expected to cause a reduction in c-kit levels.

Our results displayed a significant increase in the expression of GDF-9 in the unfertilized mature oocytes of PCOS patients after administration of NAC compared to MET, indicating that NAC, as an anti-oxidant/antiapoptotic agent, could enhance the expression of GDF-9 through inhibiting the activity of NF-kB and AP-1 transcription factors, therefore affecting the activity of MAPkinase signaling and related genes expression (33), which may be able to alleviate PCOS follicular disorders and prevent follicular developmental detention and atresia.

Our study, in agreement with aforementioned studies, also showed a significant decrease in the expression of c-kit in the oocytes of PCOS patients and also the soluble c-kit protein in the FF following administration of NAC compared to control. In addition, evidence has indicated the relationship between KL/c-kit system with MAPK pathway and/or PI3K/ Akt pathway, which are both necessary for follicle development (34). It is likely that NAC decreases the expression of c-kit through interference in MAPK pathway, all of this could be the underlying reason in the role of NAC in preventing follicular developmental detention and atresia and alleviation of follicular disorders in PCOS patients.

Although the FF content may be an invaluable hallmark for PCOS diagnosis, but the NAC ability to modulate these intra-ovarian factors of the oocyte may have interesting pharmacological perspectives for clinical management of PCOS patients. According to literature (7, 8, 32), there is an inverse relationship between c-kit and GDF-9. Therefore, improves expression of GDF-9 by NAC treatment, is expected to follow by a reduction in c-Kit and indeed MET appears to mask this effect of NAC, how this masking effect is performed, remains to be elucidated.

In this regard it has been shown that with increased follicular size and E2 production, the amount of soluble c-kit protein in human FF also increase (35), which is consistent with the correlation observed in this study between soluble c-kit with the FF volume, E2, and androstenedione concentrations.

It has been demonstrated that the excess secretion of anti-mullerian hormone (AMH) in the FF of PCOS patients may directly inhibit the production of OSFs such as GDF-9 and BMP-15, which can explain the low levels of OSFs in PCOS oocytes (36). Our findings showed a reduction in the AMH level in the FF of NAC treated group (18). Although this reduction was not statistically significant but may be considered as an underlying reason for the increased levels of GDF-9 in the NAC treated patients. Moreover, in agreement with other findings (8, 9), our study revealed no significant difference in the level of BMP-15 mRNA among the studies groups.

## Conclusion

Considering the fact that NAC improves oocyte maturation and embryo quality, and decreases the rate of immature oocytes in women with PCOS while being a safe and welltolerated agent, we suggest the administration of NAC as an alternative to other insulin-sensitizing agents like MET. Therefore, the present study argues that NAC possibly improves the oocyte quality of PCOS patients compared to MET through modulating the c-kit and GDF-9 expression, indicating that NAC supplement may be a therapeutic alternative to the insulin-sensitizing agents in PCOS management.
